# A HiBiT-tagged pseudovirus-like particle platform for safe, rapid quantification of virus neutralization and antibody-dependent enhancement

**DOI:** 10.1128/jvi.00991-25

**Published:** 2025-10-08

**Authors:** Jonathan K. Mitchell, Vincent Mastrodomenico, Jim Hartnett, William J. Heelan, Denise Garvin, Mei Cong, Jamison J. Grailer

**Affiliations:** 1 Promega Corporation 5240 , Madison, Wisconsin, USA; The Ohio State University, Columbus, Ohio, USA

**Keywords:** neutralization, bioassay, pseudovirus, antibody-dependent enhancement, NanoBiT, HiBiT, SARS-CoV-2, HIV, Ebola, monoclonal antibodies

## Abstract

Accurate quantification of virus neutralization is essential for evaluating the efficacy of vaccines and antibody-based therapies. However, conventional neutralization assays require strict biocontainment or utilize surrogates of infection that increase biosafety but reduce biological relevance. These limitations create critical bottlenecks for the timely development of antiviral immunotherapeutics. Here, we describe a neutralization platform using non-replicating, HiBiT-tagged pseudovirus-like particles (HiBiT-PsVLPs) for safe yet biologically relevant assessment of neutralization. These HiBiT-PsVLPs package the HiBiT protein tag internally and incorporate glycoproteins from pathogenic viruses to recapitulate entry at reduced biosafety levels. Unlike traditional pseudoviruses, HiBiT-PsVLPs lack reporter genes and employ the NanoBiT split luciferase system for rapid, complementation-based luminescent readout of entry and neutralization. Using the SARS-CoV-2 Spike protein as proof-of-concept, we demonstrate efficient pseudotyping, entry, and neutralization of HiBiT-PsVLPs. HiBiT-PsVLP neutralization is specific, reproducible, and reflective of antibody potency and stability. Assay results align closely with an established surrogate virus neutralization test (sVNT), with HiBiT-PsVLPs capturing more diverse mechanisms-of-action (MoAs). Moving beyond SARS-CoV-2, we adapt the HiBiT-PsVLP platform for other clinically relevant viruses, including HIV-1 and emerging pathogens, such as Ebola, Marburg, Lassa, and Nipah viruses. By pairing HiBiT-PsVLPs with Fc gamma receptor (FcγR)-expressing cells, we also demonstrate the capability of this system to detect antibody-dependent enhancement (ADE) of virus entry as an important safety consideration for immunotherapies. Combined, these data establish the utility of the HiBiT-PsVLP platform for safely and rapidly measuring critical antibody activities across diverse viruses and drug development stages.

**IMPORTANCE:**

Standard neutralization assays are often slow, labor-intensive, and restricted to high-containment facilities, thus complicating and delaying the development of vaccines and antibody-based treatments. Here, we present a novel neutralization assay system using HiBiT-tagged pseudovirus-like particles (HiBiT-PsVLPs). These particles incorporate entry proteins from diverse pathogenic viruses but are non-replicating and lack viral nucleic acids, thus mitigating the biosafety risks of conventional assays. The particles encapsulate the HiBiT peptide, enabling rapid, luminescent quantitation of entry and neutralization. We demonstrate that this platform accurately measures neutralizing activity of monoclonal antibodies across development stages and sensitively detects antibody-dependent enhancement, a critical safety consideration. Altogether, HiBiT-PsVLPs offer a safe, rapid, and scalable platform to accelerate the development of vaccines and antibody therapeutics targeting a broad range of viruses.

## INTRODUCTION

Viral disease outbreaks are increasing in frequency alongside rising global connectivity, urbanization, climate change, and other socio-economic, environmental, and ecological factors (1). Over the past decade alone, the world has experienced multiple epidemics, including the 2014–2016 Ebola epidemic in Western Africa, the Zika epidemic in the Americas (2015–2016), the COVID-19 pandemic, and the Mpox global health emergency. Amidst these emerging virus outbreaks, established pathogens, such as human immunodeficiency virus (HIV), hepatitis viruses, and influenza viruses, remain major causes of global morbidity and mortality (2–4). New countermeasures are thus needed to combat both endemic and emerging viral threats.

Monoclonal antibodies (mAbs) have arisen as powerful, dual-modality interventions with niche roles in antiviral defense (5). By virtue of their high affinity and specificity, mAbs can serve as potent prophylactics and therapeutics with minimal off-target effects. As prophylactics, mAbs confer immediate protection against infection, providing an important stopgap for vaccine-induced immunity in the nascent stages of an outbreak (6). Such protection is especially critical for populations with high exposure risk, including healthcare professionals and close contacts of infected individuals. MAbs may also serve as supplements or alternatives to vaccination for immunocompromised individuals unlikely to mount a protective vaccine response (7). As therapeutics, mAbs can decrease infectious viral loads, reduce the risk of hospitalization and death, and potentially lower the likelihood of transmission (6).

Like antibodies elicited by vaccination or infection, mAbs exert their antiviral activity via immune effector functions (e.g., antibody-dependent cellular cytotoxicity) and/or virus neutralization (8). Neutralizing antibodies (nAbs) bind specifically to viral epitopes essential for receptor binding, membrane fusion, endosomal escape, or genome uncoating, thereby inhibiting virus entry. This ability to block infection underpins the prophylactic and therapeutic efficacy of neutralizing monoclonal antibodies (NMAbs). Despite their antiviral properties, however, virus-specific antibodies can paradoxically exacerbate disease in certain contexts. In its best-understood form, this antibody-dependent enhancement (ADE) occurs when non- or sub-neutralizing antibodies bind virus particles and engage Fc gamma receptors (FcγRs) on phagocytes, increasing viral uptake, entry, and replication (9). Because neutralization and ADE are crucial to NMAb efficacy and safety, robust, mechanism-of-action (MoA)-reflective methods for measuring these processes are vital for NMAb development.

A variety of methods currently exist for assessing neutralization and ADE, each with distinct advantages and limitations (10). Live virus assays, such as the plaque reduction neutralization test, are typically considered the “gold standard” for their biological relevance. However, these assays are intrinsically low-throughput and require high biocontainment for pathogenic viruses and multiple days for readout. Surrogate methods are therefore commonly deployed, including enzyme-linked immunosorbent assay (ELISA)-based surrogate virus neutralization tests (sVNTs) and pseudovirus neutralization assays (PNAs). sVNTs measure interactions between purified viral receptor binding domains (RBDs) and their cognate receptors (11). Although fast and free of biosafety constraints, these assays feature several key drawbacks: (i) they do not reflect the full complexity of virus entry, (ii) they capture only those nAbs that disrupt RBD-receptor binding, and (iii) they cannot detect ADE. By comparison, PNAs offer a balance between biosafety and biological relevance by using replication-incompetent viruses bearing the entry protein(s) from the virus of interest (12). These pseudoviruses recapitulate virus entry into host cells, allowing for the detection of more diverse antibody MoAs than sVNTs. Reporter genes, such as those encoding fluorescent proteins or luciferases, may be packaged within pseudoviruses for quantitative assessment of neutralization and ADE. Despite these advantages, PNAs nonetheless carry biosafety risks, including the potential for replication-competent virus production and insertional mutagenesis (i.e., for lentivirus-based pseudoviruses) (13, 14). Additionally, PNAs require one or more days for readout (15).

To overcome the limitations of existing assays, we utilized the HiBiT protein tagging system to develop a safe, biologically relevant method to rapidly quantify neutralization and ADE. HiBiT is an 11 amino acid peptide tag that binds to its complementary polypeptide LgBiT with high affinity to reconstitute functional NanoBiT luciferase (16). By fusing HiBiT to the HIV-1 Pr55^Gag^ polyprotein, we derive enveloped VLPs that package the HiBiT peptide internally. This highly modular VLP platform can be pseudotyped with diverse viral glycoproteins to permit cell entry. Pairing these HiBiT-tagged pseudovirus-like particles (HiBiT-PsVLPs) with LgBiT-expressing cells yields a luminescent assay system with improved biosafety and reduced turnaround times relative to conventional PNAs. Unlike sVNTs, HiBiT-PsVLP bioassays capture MoAs beyond inhibition of RBD-receptor binding and enable detection of both neutralization and ADE. Here, we establish the feasibility and robustness of the HiBiT-PsVLP platform and demonstrate its broad applicability for NMAb development against endemic and emerging viruses.

## RESULTS

### Production and characterization of SARS-CoV-2 S HiBiT-PsVLPs

**Fig 1:**
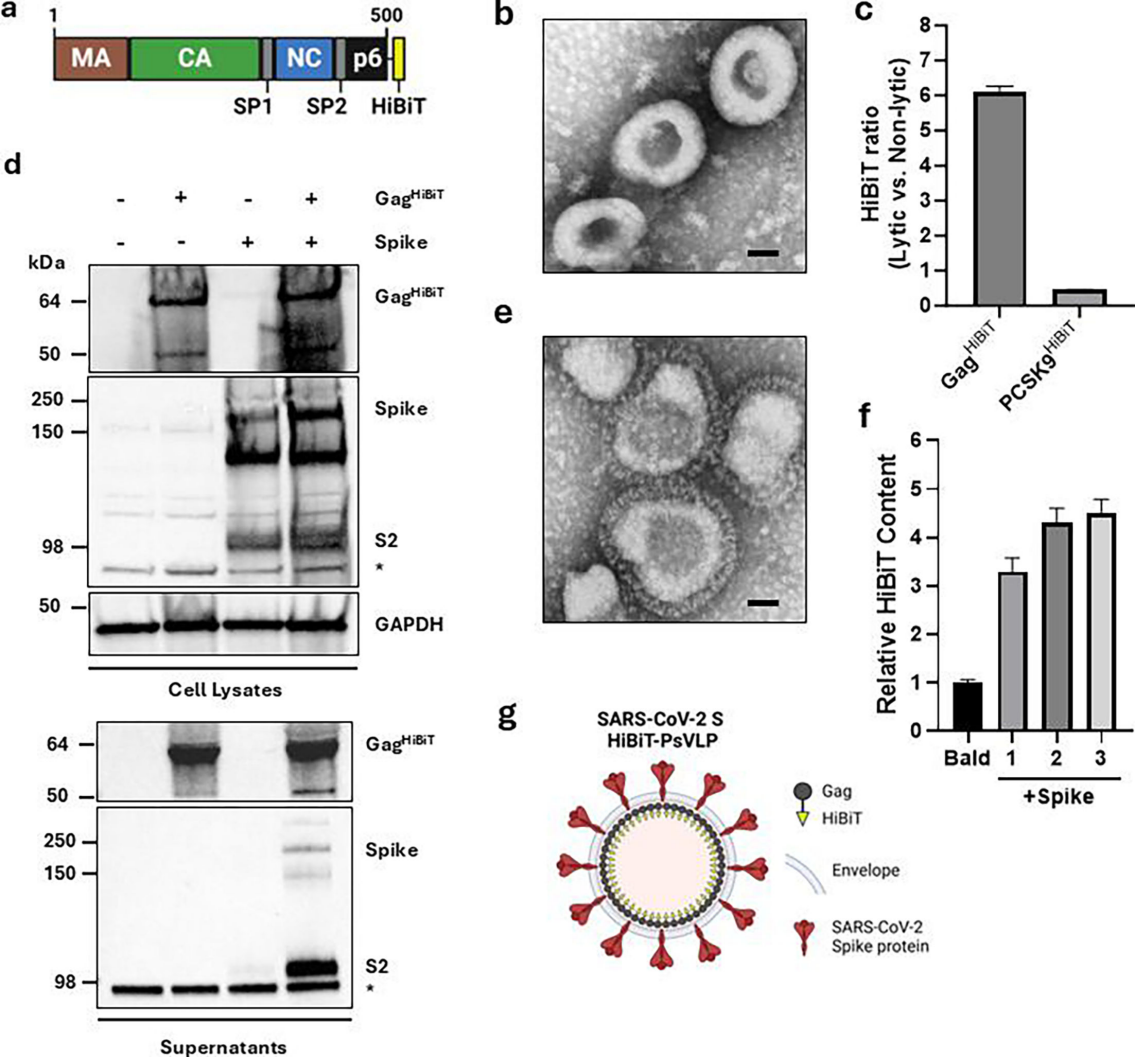
Production and characterization of SARS-CoV-2 S HiBiT-PsVLPs. (**a**) Schematic representation of the Gag^HiBiT^ polyprotein indicating matrix (MA), capsid (CA), spacer peptides (SP1/SP2), and p6 region along with the C-terminal HiBiT tag. Numbers denote amino acid positions within the Gag polyprotein. (**b**) Electron micrograph of purified Gag^HiBiT^ VLPs. Scale bar = 50 nm. (**c**) HiBiT packaging within VLPs, as measured via lytic vs non-lytic HiBiT detection in culture supernatants of 293T cells transfected with Gag^HiBiT^ or PCSK9^HiBIT^. (**d**) Immunoblot analysis of Gag^HiBiT^ and SARS-CoV-2 Spike protein in lysates (top) and culture supernatants (bottom) from transfected 293T cells. Bands corresponding to full-length Gag^HiBiT^, full-length Spike protein, and the Spike S2 subunit are indicated. *, Non-specific protein band detected by anti-Spike S2 antibody. GAPDH was used as a loading control for cell lysates. (**e**) Electron micrograph of purified SARS-CoV-2 S HiBiT-PsVLPs. Scale bar = 50 nm. (**f**) HiBiT content per particle in Bald vs SARS-CoV-2 S HiBiT-PsVLPs. Relative HiBiT content was calculated as the ratio of HiBiT to p24 for a reference batch of Bald HiBiT-VLPs and three independent batches of SARS-CoV-2 S HiBiT-PsVLPs. Ratios were normalized to Bald HiBiT-VLPs. (**g**) Cartoon representation of SARS-CoV-2 S HiBiT-PsVLPs with Gag^HiBiT^, membrane envelope, and SARS-CoV-2 Spike protein indicated. Image created with BioRender.com.

To generate HiBiT-tagged VLPs, we fused HiBiT to the C-terminus of HIV-1 Pr55^Gag^ (Fig. 1a). Transient expression of this Gag^HiBiT^ fusion protein yielded electron-dense structures in culture supernatants consistent in size (~100 nm) and morphology with HIV-1 Gag VLPs (Fig. 1b) (17, 18). HiBiT detection assays indicated successful packaging of Gag^HiBiT^ within enveloped VLPs, in contrast to a secreted, non-enveloped control protein (PCSK9^HiBiT^) (Fig. 1c).

We next sought to pseudotype Gag^HiBiT^ VLPs with the SARS-CoV-2 Spike protein. To this end, we co-expressed Gag^HiBiT^ with truncated Spike protein lacking the cytoplasmic tail, as removal of this endodomain has been shown to improve pseudotyping (19). Immunoblotting confirmed Spike expression and processing, which were unaffected by Gag^HiBiT^ co-expression (Fig. 1d). Minimal Spike secretion was observed in the absence of Gag^HiBiT^, whereas secretion, primarily of processed Spike, was enhanced by Gag^HiBiT^ co-expression. Electron microscopy revealed VLPs with distinctive crown-like halos characteristic of coronavirus Spike proteins (Fig. 1e). HiBiT content per particle was similar across three independent batches of SARS-CoV-2 S HiBiT-PsVLPs and was increased relative to non-pseudotyped, Bald HiBiT-VLPs, indicating that Spike incorporation did not reduce HiBiT packaging efficiency (Fig. 1f). Combined, these data demonstrate successful production of enveloped Gag^HiBiT^ VLPs pseudotyped with SARS-CoV-2 Spike protein (Fig. 1g).

### Cell entry by SARS-CoV-2 S HiBiT-PsVLPs

**Fig 2:**
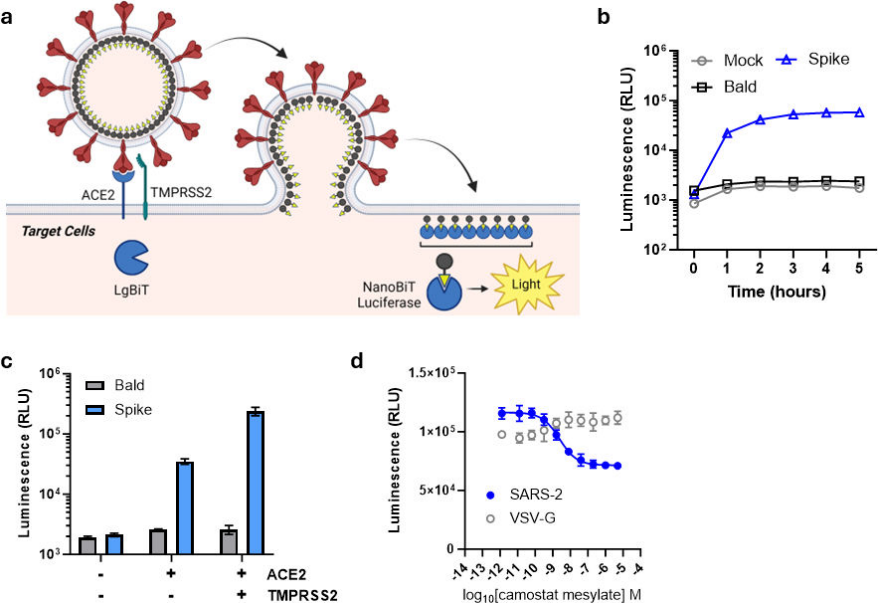
SARS-CoV-2 S HiBiT-PsVLP entry into SARS-CoV-2 HEK293T(LgBiT) target cells. (**a**) Model for SARS-CoV-2 S HiBiT-PsVLP entry. SARS-CoV-2 Spike mediates attachment of HiBiT-PsVLPs to ACE2 receptor expressed on the target cells. TMPRSS2 proteolytically activates Spike, resulting in membrane fusion and exposure of Gag^HiBiT^ within LgBiT-expressing target cells. HiBiT and LgBiT undergo protein complementation to form NanoBiT luciferase, yielding a luminescent signal in the presence of substrate. Image created with BioRender.com. (**b**) Time course of HiBiT-PsVLP entry. Mock, target cells alone. Bald, HiBiT-PsVLPs lacking viral entry protein. Spike, SARS-CoV-2 S HiBiT-PsVLPs. (**c**) HiBiT-PsVLP entry into LgBiT Target Cells ± ACE2 and TMPRSS2 expression. (**d**) Inhibition of SARS-CoV-2 S HiBiT-PsVLP entry by TMPRSS2 inhibitor, camostat mesylate. VSV-G HiBiT-PsVLPs were used as a control for TMPRSS2-independent entry.

To measure entry and neutralization of SARS-CoV-2 S HiBiT-PsVLPs, we generated a clonal 293T target cell line stably expressing LgBiT, SARS-CoV-2 receptor ACE2, and the Spike-activating protease TMPRSS2 (Fig. S1) (20). SARS-CoV-2 S HiBiT-PsVLP entry into these target cells is predicted to culminate in NanoBiT luciferase complementation and luminescence (Fig. 2a). Indeed, the addition of SARS-CoV-2 S HiBiT-PsVLPs to target cells resulted in a luminescent signal that increased rapidly from 0 to 2 h and plateaued by 3 to 4 h (Fig. 2b). HiBiT-PsVLP entry was Spike-dependent, as Bald HiBiT-VLPs yielded similar luminescence to target cells alone. SARS-CoV-2 S HiBiT-PsVLP entry was ACE2-dependent and enhanced by TMPRSS2 expression (Fig. 2c). Conversely, TMPRSS2 inhibition by camostat mesylate reduced SARS-CoV-2 S HiBiT-PsVLP entry into ACE2^+^/TMPRSS2^+^ target cells but had no effect on entry of HiBiT-PsVLPs pseudotyped with Vesicular Stomatitis Virus glycoprotein (VSV-G) (Fig. 2d). Collectively, these results demonstrate rapid luminescent detection of SARS-CoV-2 Spike-mediated cell entry using the HiBiT-PsVLP system.

### Neutralization of SARS-CoV-2 S HiBiT-PsVLPs by anti-Spike NMAbs

**Fig 3:**
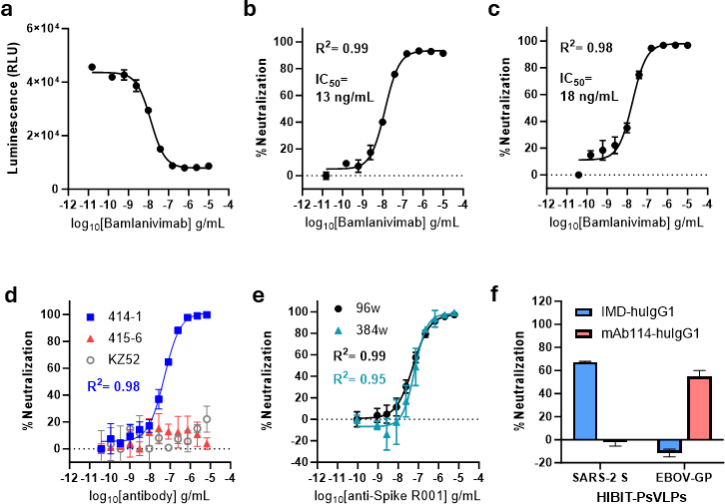
Neutralization of SARS-CoV-2 S HiBiT-PsVLPs. (**a and b**) Neutralization of SARS-CoV-2 S HiBiT-PsVLPs by bamlanivimab biosimilar using freshly cultured SARS-CoV-2 HEK293T(LgBiT) target cells. Data are represented as (**a**) raw luminescence and (**b**) percent neutralization. (**c**) Neutralization of SARS-CoV-2 S HiBiT-PsVLPs by bamlanivimab biosimilar using thaw-and-use SARS-CoV-2 HEK293T(LgBiT) target cells. (**d**) Specificity of SARS-CoV-2 S HiBiT-PsVLP neutralization as assayed using anti-Spike NMAb clone 414-1, non-neutralizing anti-Spike mAb clone 415-6, or anti-Ebola virus glycoprotein (EBOV GP) NMAb clone KZ52. (**e**) SARS-CoV-2 S HiBiT-PsVLP neutralization bioassay performance in 96- vs 384-well format. (**f**) Target-specific neutralization of HiBiT-PsVLPs by transiently expressed NMAbs in culture supernatants. HiBiT-PsVLPs were challenged with culture supernatants from 293T cells transfected with anti-Spike NMAb (IMD-huIgG1) or anti-EBOV GP NMAb (mAb114-huIgG1) expression constructs.

Antibody neutralization of SARS-CoV-2 S HiBiT-PsVLPs was first assessed using freshly cultured target cells and a biosimilar of anti-Spike NMab bamlanivimab (21). Given the entry kinetics observed in Fig. 2b, a neutralization assay endpoint of 3 h was chosen. Bamlanivimab biosimilar reduced assay luminescence in a concentration-dependent manner, fully neutralizing SARS-CoV-2 S HiBiT-PsVLPs at the highest concentrations tested (Fig. 3a and b). Similar results were obtained using cryopreserved, thaw-and-use target cells, which expressed ACE2 and TMPRSS2 at levels comparable to freshly cultured cells (Fig. 3c; Fig. S1c). Thaw-and-use cells (commonly referred to as ready-to-use or assay-ready cells) offer distinct advantages over continuous cell culture, including greater assay reproducibility, cost/time savings, and flexibility in scheduling assays (22). As such, thaw-and-use target cells were utilized for neutralization assays throughout the remainder of this study.

Specificity of SARS-CoV-2 S HiBiT-PsVLP neutralization was evaluated using anti-Spike mAb clones previously isolated from convalescent patients (23). We selected a HiBiT-PsVLP input of ~1–2 × 10^5^ RLUs per well, which yields >5-fold signal-to-background and falls within a robust operating range for neutralization (Fig. S2). This input was used for all subsequent neutralization experiments. NMAb clone 414-1 neutralized SARS-CoV-2 S HiBiT-PsVLPs, whereas the non-neutralizing anti-Spike clone 415-6 had no effect (Fig. 3d). KZ52, an NMAb targeting the Ebola virus glycoprotein (EBOV GP), also failed to neutralize SARS-CoV-2 S HiBiT-PsVLPs, further demonstrating specificity for anti-Spike NMAbs (24).

To assess compatibility with early-stage NMAb discovery workflows, we compared assay performance in 96- vs 384-well plate formats. Raw luminescence values decreased in 384-well format, as expected given reduced assay volumes (data not shown). Nonetheless, HiBiT-PsVLP neutralization aligned closely between formats, confirming the assay can be miniaturized for high-throughput workflows (Fig. 3e).

During NMAb discovery, candidate NMAbs are routinely expressed in mammalian cells, and mAb-containing supernatants are screened for neutralizing activity (25). To simulate this approach, we generated human IgG1 expression constructs bearing the variable regions of anti-Spike NMAb imdevimab (IMD-huIgG1) or the anti-EBOV GP NMAb mAb114 (mAb114-huIgG1) (26, 27). Culture supernatants from cells transfected with these constructs were assayed for neutralization of SARS-CoV-2 S and EBOV GP HiBiT-PsVLPs (Fig. 3f). Supernatant from IMD-huIgG1 expressing cells neutralized SARS-CoV-2 S but not EBOV GP HiBiT-PsVLPs. Conversely, supernatant from mAb114-huIgG1 expressing cells neutralized EBOV GP but not SARS-CoV-2 S HiBiT-PsVLPs. These results confirm viral glycoprotein-specific neutralization of HiBiT-PsVLPs by transiently expressed NMAbs in culture supernatants.

### Qualification of the SARS-CoV-2 HiBiT-PsVLP bioassay

**Fig 4:**
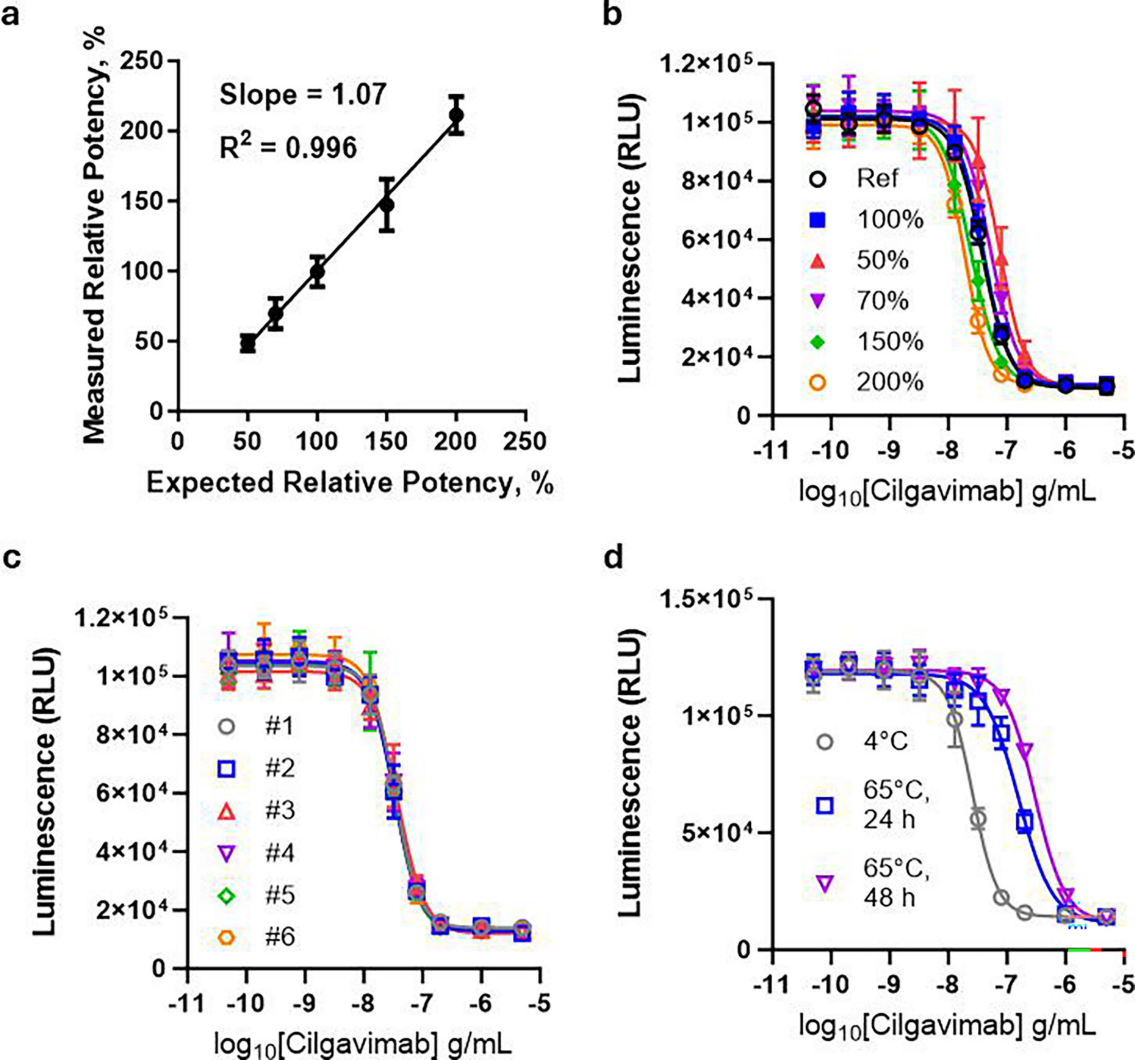
Qualification of the SARS-CoV-2 HiBiT-PsVLP bioassay. Assays were performed using a biosimilar of anti-Spike NMAb cilgavimab. (**a and b**) Assay linearity. (**a**) Expected vs measured relative potency values from three independent assay runs, each by two separate analysts. (**b**) Results from a representative assay run comparing NMAb activity at the indicated relative potencies vs a reference standard (Ref). (**c**) Intra-assay repeatability determined from six independent NMAb titrations. (**d**) Stability of heat-stressed NMAb samples incubated at 4°C or at 65°C for the indicated lengths of time before performing the SARS-CoV-2 HiBiT-PsVLP bioassay.

The SARS-CoV-2 S HiBiT-PsVLP neutralization bioassay was qualified in accordance with International Conference on Harmonization of Technical Requirements for Pharmaceuticals for Human Use Q2(R2) guidelines (28). Results are summarized in Table 1 and Fig. 4. Assay specificity was confirmed in Fig. 3d. The assay was linear (R^2^ = 0.996) across a range of 50% to 200% relative potency (Fig. 4a). Figure 4b shows dose-response curves from a representative assay comparing NMAb samples at 50%, 70%, 100%, 150%, and 200% relative potency. Repeatability (*n* = 6) was 3.9% (Fig. 4c). Intermediate precision was 11.2%. Assay accuracy across the different relative potencies ranged from 96.8% to 105.6% (Table 1).

**TABLE 1:** Qualification summary for the SARS-CoV-2 HiBiT-PsVLP bioassay^*a*^

Parameter	Results		
	Measured value	Expected relative potency	Recovery, %
Accuracy	48.4%	50%	96.8%
	69.7%	70%	99.6%
	99.5%	100%	99.5%
	147%	150%	98.1%
	211%	200%	106%
Repeatability (% CV)	3.9%	100% (reference)	
Intermediate precision (% CV)	11.2%		
Linearity (R^2^)	0.996		
Linearity (y = mx + b)	Y = 1.07 x − 6.38		

^*a*^ Values represent aggregate data from three independent assays, each performed by two separate analysts (*n* = 6 total assays).

Stability-indicating properties of the assay were tested using heat-stressed NMAb samples. The assay detected a loss of NMAb potency with increasing incubation time at 65°C relative to an unstressed sample maintained at 4°C (Fig. 4d). Relative potencies decreased by ~83% and ~91% for samples incubated at 65°C for 24 or 48 h, respectively, confirming that the assay is stability-indicating. Together, these results demonstrate that the analytical performance of this bioassay is appropriate for quality testing of NMAbs in accordance with Good Manufacturing Practices (GMP).

### Benchmarking against a surrogate virus neutralization test

**Fig 5:**
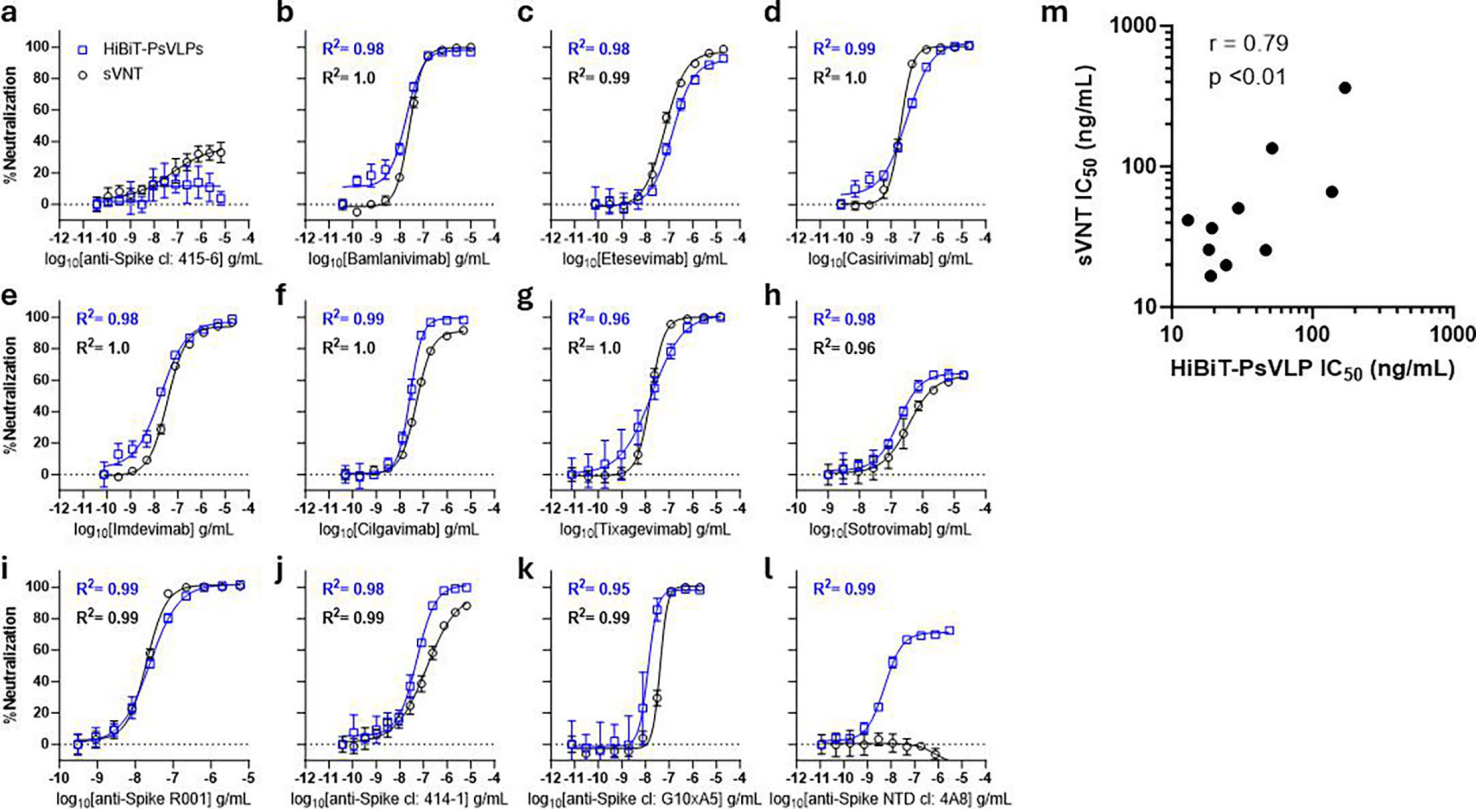
Assay comparison for SARS-CoV-2 HiBiT-PsVLPs vs sVNT. (**a–l**) Neutralizing activity of anti-Spike mAbs as measured using the SARS-CoV-2 HiBiT-PsVLP bioassay (HiBiT-PsVLPs) vs sVNT. (**a**) Non-neutralizing anti-Spike mAb clone 415-6 was used as a negative control for both assays. (**b–k**) anti-Spike RBD NMAbs. (**l**) anti-Spike N-terminal domain (NTD) NMAb clone 4A8. R^2^ values in blue are from the SARS-CoV-2 HiBiT-PsVLP bioassay. Values in black are from the sVNT. (**m**) Correlation of anti-Spike RBD NMAb IC_50_ values (ng/mL) derived from the SARS-CoV-2 HiBiT-PsVLP bioassay vs sVNT. R, Pearson correlation coefficient.

sVNTs are an established method commonly used to assess antibody neutralization without the need for virus or pseudovirus (10, 11). Like HiBiT-PsVLPs, sVNTs largely alleviate biosafety concerns and provide a rapid and quantitative measurement of NMAb activity. Given these similarities, we sought to compare the SARS-CoV-2 HiBiT-PsVLP bioassay with a commercially available sVNT using a panel of anti-Spike mAbs (11). Non-neutralizing anti-Spike clone 415-6 was used as a negative control and showed minimal activity in both tests (Fig. 5a). Conversely, NMAbs targeting the Spike RBD exhibited concentration-dependent neutralizing activity in both assays, yielding sigmoidal, 4-parameter logistic dose-response curves with R^2^ values ≥0.95 (Fig. 5b through k). Neutralization potencies, expressed as half-maximal inhibitory concentrations (IC_50_ values), were determined for each of these NMAbs and compared between assays. A strong positive correlation (Pearson r = 0.79) was observed between the HiBiT-PsVLP bioassay and the sVNT (Fig. 5m).

Notably, NMAb clone 4A8 targeting the Spike N-terminal domain (NTD) exhibited neutralizing activity exclusively in the HiBiT-PsVLP bioassay (Fig. 5l). This result was expected, as the sVNT includes only the purified Spike RBD, whereas HiBiT-PsVLPs incorporate the full ectodomain and transmembrane region of Spike. Thus, while highly correlative to the sVNT for RBD-specific NMAbs, the HiBiT-PsVLP bioassay offers broader epitope coverage and detection of more diverse MoAs.

### HiBiT-PsVLPs for anti-HIV NMAb development

Given the inherent flexibility of pseudotyping, we predicted that the HiBiT-PsVLP platform could be adapted for other clinically relevant viruses. Despite a sharp decline in HIV-related deaths since their peak in 2004, more than 500,000 such deaths still occur annually (2). Thus, there is a continued need for new and improved countermeasures against HIV, including NMAbs and other prophylactics capable of reducing virus transmission.

**Fig 6:**
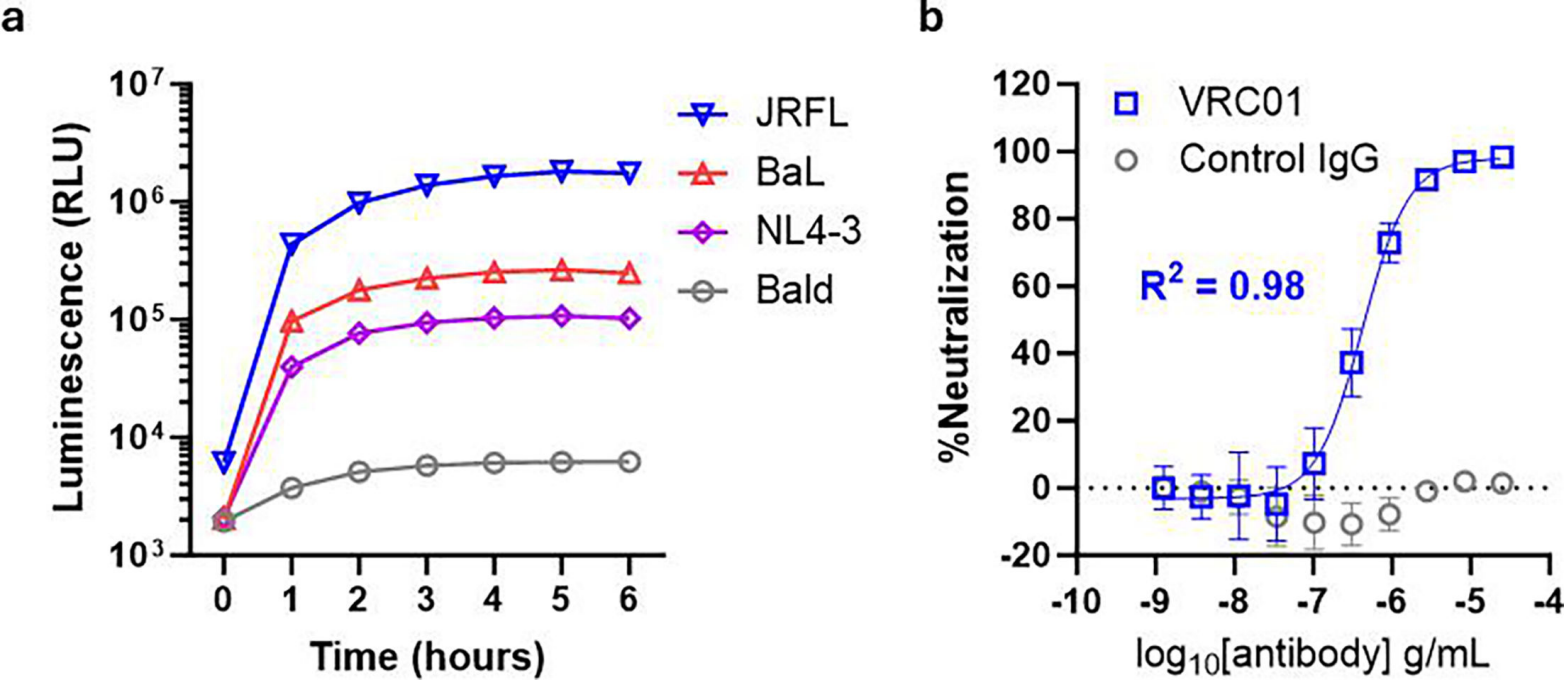
Entry and neutralization of HIV Env HiBiT-PsVLPs. (**a**) Time course for HiBiT-PsVLP entry into HIV HEK293T(LgBiT) target cells. Cells were overlaid with HiBiT-PsVLPs bearing HIV Env protein from the indicated isolates or Bald HiBiT-VLPs lacking entry protein. (**b**) Neutralization of HIV Env(JRFL) HiBiT-PsVLPs by anti-HIV gp120 NMAb VRC01 vs isotype-matched control IgG.

HIV-1 neutralization is commonly assessed using the TZM-bl assay, a specialized PNA involving transactivation of cellular reporter genes by pseudovirus-encoded HIV Tat protein (29). Although this assay offers improved biosafety compared to replication-competent HIV-1, it nonetheless requires multiple days for readout. We therefore reasoned that HiBiT-PsVLPs could provide a faster method for assaying HIV-1 neutralization. To test this prediction, we first produced a 293T target cell line stably expressing LgBiT along with the HIV-1 receptor CD4 and co-receptors CCR5 and CXCR4 (Fig. S3). We next generated HiBiT-PsVLPs bearing the HIV-1 envelope (Env) protein from two different CCR5-tropic isolates, BaL and JRFL, and one CXCR4-tropic strain, NL4-3. The endodomain of each HIV Env protein was truncated to improve pseudotyping (30). Addition of HIV Env HiBiT-PsVLPs to target cells resulted in increased luminescence over time, with peak entry observed by 4 to 5 h (Fig. 6a).

For neutralization studies, we utilized HiBiT-PsVLPs bearing Env protein from the JRFL isolate. This isolate exhibits a tier 2 neutralization phenotype considered typical of most circulating HIV-1 strains (31, 32). HIV Env(JRFL) HiBiT-PsVLPs were challenged with VRC01, a potent anti-HIV gp120 NMAb that targets the CD4 binding site and neutralizes ~90% of HIV-1 isolates (33). Based on results from Fig. 6a, a 4 h assay endpoint was selected. HIV Env(JRFL) HiBiT-PsVLPs were neutralized by VRC01, whereas a non-specific control antibody had no impact on entry (Fig. 6b). These data demonstrate the feasibility of using HiBiT-PsVLPs to probe HIV-1 neutralization with dramatically reduced turnaround times relative to the TZM-bl assay.

### HiBiT-PsVLPs for emerging viral pathogens

The World Health Organization and Coalition for Epidemic Preparedness Innovations have identified panels of key emerging viruses to guide global pandemic preparedness efforts (34, 35). These priority pathogens were selected based on their epidemic and pandemic potential, their high rates of morbidity and mortality, and the lack of specific countermeasures against them. As many of these viruses are Risk Group four agents, we predicted that the HiBiT-PsVLP platform could provide a safe yet biologically relevant surrogate for assessing their neutralization. To address this hypothesis, we first generated HiBiT-PsVLPs bearing the glycoprotein (GP) from three distinct filoviruses: Zaire Ebola virus (EBOV), Sudan Ebola virus (SEBOV), or Marburg virus (MARV), each of which causes severe hemorrhagic fever in humans. Despite licensed vaccines and NMAbs for EBOV, no specific countermeasures currently exist for MARV or SEBOV.

**Fig 7:**
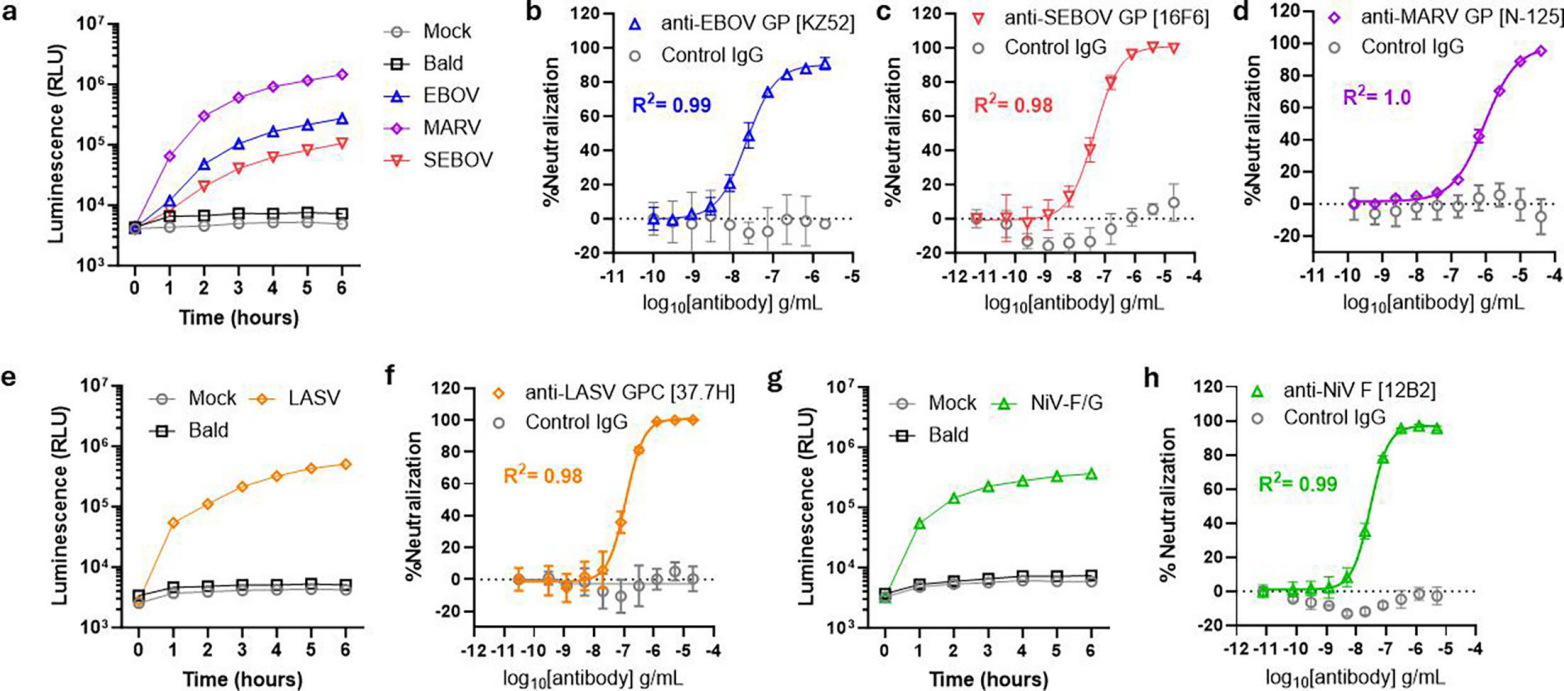
Entry and neutralization of HiBiT-PsVLPs for priority viral pathogens. (**a**) Entry time course for HiBiT-PsVLPs bearing the indicated filovirus GP. EBOV, Zaire Ebola virus. MARV, Marburg virus. SEBOV, Sudan Ebola virus. (**b–d**) Neutralization of filovirus GP HiBiT-PsVLPs by the indicated NMAbs. (**b**) EBOV GP, (**c**) SEBOV GP, and (**d**) MARV GP. (**e**) Entry time course for Lassa virus (LASV) glycoprotein complex (GPC) HiBiT-PsVLPs. (**f**) Neutralization of LASV-GPC HiBiT-PsVLPs. (**g**) Entry time course for NiV-F/G HiBiT-PsVLPs. (**h**) Neutralization of NiV-F/G HiBiT-PsVLPs. Isotype-matched IgG was used as a negative control for each NMAb.

Entry of filovirus GP HiBiT-PsVLPs was confirmed using LgBiT-expressing 293T cells (Fig. 7a). Filovirus GP-mediated entry was notably slower than that driven by SARS-CoV-2 S or HIV Env, consistent with differences in viral entry pathways (Fig. S4). Whereas SARS-CoV-2 S and HIV Env can mediate entry at the plasma membrane, filovirus GPs require trafficking to late endosomes and/or lysosomes for binding to the intracellular receptor NPC-1 and subsequent membrane fusion (36–38). This additional trafficking step likely delays luminescence onset in the HiBiT-PsVLP assay. Nonetheless, each filovirus HiBiT-PsVLP was neutralized by an NMAb targeting its GP (Fig. 7b through d), demonstrating specific neutralization despite pathway-dependent differences in entry kinetics.

We further extended the HiBiT-PsVLP platform to two other priority pathogens: (i) Lassa virus (LASV), an arenavirus causing acute hemorrhagic illness, and (ii) Nipah virus (NiV), a paramyxovirus causing acute respiratory disease and fatal encephalitis. LgBiT-expressing 293T cells were selected as the target cell line, as they endogenously express the primary LASV receptor, α-dystroglycan, and NiV receptors, EphrinB2 and EphrinB3 (39–42). HiBiT-PsVLPs bearing the LASV glycoprotein complex (GPC) entered target cells and were neutralized specifically by an anti-LASV GPC NMAb (Fig. 7e and f). NiV HiBiT-PsVLPs were generated using the viral attachment glycoprotein (G) and an endodomain-truncated viral fusion protein (F) (43). These HiBiT-PsVLPs entered target cells and were neutralized by an anti-NiV F NMAb (Fig. 7g and h). Together, these studies demonstrate the flexibility of the HiBiT-PsVLP platform and its potential to facilitate NMAb development across a variety of emerging viral pathogens.

### Measuring antibody-dependent enhancement with HiBiT-PsVLPs

**Fig 8:**
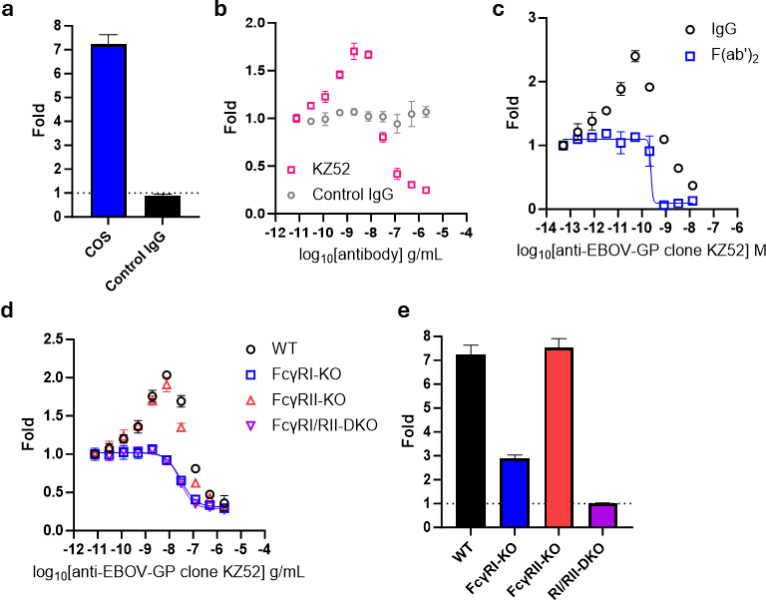
ADE of EBOV GP HiBiT-PsVLP entry. (**a**) Fold enhancement of EBOV GP HiBiT-PsVLP entry by 3 µg/mL cosfroviximab (COS) biosimilar or isotype-matched control IgG relative to no antibody. (**b**) ADE assay response to increasing concentrations of anti-EBOV GP NMAb clone KZ52 vs isotype-matched control IgG. (**c**) Comparison of ADE assay response for full-length anti-EBOV GP NMAb clone KZ52 (IgG) vs F(ab′)_2_ fragment. (**d and e**) Evaluation of ADE in wild-type (WT) vs FcγR-KO THP-1/HaloTag-LgBiT target cells using (**d**) a titration of anti-EBOV GP NMAb clone KZ52 or (**e**) 3 ug/mL cosfroviximab biosimilar. DKO, double-knockout.

ADE assays typically rely on live viruses or pseudoviruses and are subject to many of the same pitfalls as conventional neutralization assays. We therefore sought to determine whether HiBiT-PsVLPs could provide a safe and rapid surrogate for measuring ADE. To this end, we engineered LgBiT expression into THP-1 human monocyte cells that endogenously express FcγRI and FcγRIIa (Fig. S5) (44, 45). We then assayed entry of EBOV GP HiBiT-PsVLPs into this target cell line in the presence of different mAbs. EBOV GP HiBiT-PsVLP entry was enhanced approximately sevenfold by cosfroviximab, a non-neutralizing anti-EBOV-GP mAb previously shown to induce ADE *in vitro* (Fig. 8a) (46). Isotype-matched control mAb lacking specificity for EBOV GP had no effect on HiBiT-PsVLP entry. In contrast to cosfroviximab, anti-EBOV-GP NMAb KZ52 yielded a bi-phasic response, enhancing entry at low concentrations and neutralizing HiBiT-PsVLPs at higher concentrations (Fig. 8b). The Fc domain of KZ52 was required for enhancement but was dispensable for neutralization, consistent with a previous report (Fig. 8c) (24).

To confirm the role of FcγRs, we generated THP-1/HaloTag-LgBiT CRISPR knockout lines lacking FcγRI, FcγRII, or both receptors (Fig. S5). ADE by cosfroviximab and KZ52 was reduced or eliminated by knockout of FcγRI, whereas FcγRII knockout had no significant effect on ADE (Fig. 8d and e). Neutralizing activity of KZ52 was preserved across the different knockout lines (Fig. 8d). Taken together, these results are consistent with the detection of classical, FcγR-associated ADE driven primarily by mAb Fc interaction with FcγRI.

## DISCUSSION

NMAbs play unique roles in antiviral defense, with potent and, ideally, broadly acting NMAbs serving as both therapeutics and prophylactics. Robust, MoA-reflective neutralization assays are essential to identify the most promising NMAbs and to shepherd these candidates through the development pipeline. In this study, we established the HiBiT-PsVLP system as a safe, fast, and versatile bioassay platform with applications spanning multiple stages of NMAb development. This platform is compatible with standard discovery workflows, enables rapid characterization of NMAb potency and stability, and demonstrates performance characteristics suitable for quality release testing. Additionally, this system can be adapted to measure ADE and thereby identify potential safety concerns at early stages of NMAb development.

Surrogate neutralization assays provide safer, higher-throughput, and faster alternatives to live virus neutralization tests and are thus better suited to many research and GMP environments. Nonetheless, each surrogate assay features distinct advantages and limitations. ELISA-based methods (e.g., sVNTs) are rapid and safe but fail to capture the full complexity of virus entry, typically measuring only the interaction between a viral RBD and its cognate receptor. Conversely, pseudoviruses incorporate the full ectodomains of viral glycoproteins and recapitulate native virus entry, thus offering greater biological relevance than sVNTs. However, PNAs are dramatically slower than sVNTs, with assay times typically ranging from 1 to 4 days. By comparison, HiBiT-PsVLPs deliver the same benefits as traditional PNAs with reduced assay times approaching those of sVNTs. Biosafety of HiBiT-PsVLPs is also improved over HIV-based pseudoviruses, as HiBiT-PsVLPs lack enzymes encoded by the HIV *pol* gene and do not rely on packaging of viral nucleic acids. These changes mitigate the risks of insertional mutagenesis and recombination to form replication-competent viruses. Although our study benchmarked HiBiT-PsVLPs against a commercial sVNT to provide a standardized reference, future work should include direct comparisons with PNAs and live virus assays to more clearly define how HiBiT-PsVLPs align with established methods.

Unlike conventional PNAs, HiBiT-PsVLP bioassays require the use of LgBiT-expressing target cells for complementation-based readout. For many widely used, “workhorse” cell lines, such as the HEK293T cells used in this study, LgBiT expression can be easily introduced via stable plasmid transfection. For more specialized models (e.g., primary cell cultures), alternative gene delivery methods, such as LgBiT mRNA transfection or viral transduction, may be necessary. In each case, the time required for LgBiT gene delivery would be at least partially offset by the reduced turnaround times of HiBiT-PsVLP bioassays relative to PNAs. Also, unlike pseudoviruses, HiBiT-PsVLPs do not provide intrinsic signal amplification through reporter gene expression, resulting in lower signal-to-background ratios. Nonetheless, luminescence and signal-to-background are sufficient to quantitatively assess neutralization and ADE, as demonstrated in this study.

Like pseudoviruses, HiBiT-PsVLPs can be rapidly adapted for different viral pathogens by exchanging the glycoprotein(s) on their surface. In our study, HiBiT:p24 ratios were elevated for SARS-CoV-2 S HiBiT-PsVLPs relative to Bald controls. Because each Gag molecule carries a single HiBiT tag, this increase is unlikely to reflect changes in HiBiT stoichiometry per Gag protein but may instead arise from differences in particle composition or detection efficiency. Importantly, the elevated ratios were similar across independent preparations, supporting the reproducibility of HiBiT incorporation. Beyond SARS-CoV-2 and HIV, we anticipate the HiBiT-PsVLP platform could be applied to other endemic viruses of global public health concern. As demonstrated here, HiBiT-PsVLPs can also be adapted for many priority viruses of epidemic/pandemic potential, highlighting the utility of this platform for pandemic preparedness. HiBiT-PsVLPs could also be leveraged for mutagenesis studies of viral glycoproteins to identify mutations that alter NMAb sensitivity. Similarly, these assays could be utilized in the discovery and development of NMAb cocktails designed to reduce the likelihood of mutational escape.

As with PNAs, the current HiBiT-PsVLP platform is limited to enveloped viruses. Additionally, glycoproteins from some enveloped viruses, such as Dengue virus, are difficult to incorporate into heterologous virions and may likewise prove incompatible with our HIV-1 Gag-based system (47). In such cases, the HiBiT tag can be appended to structural proteins derived from the same virus or related family members to generate HiBiT-tagged VLPs capable of incorporating these glycoprotein(s) (48). The HiBiT-VLP approach was also recently extended to non-enveloped viruses by fusing the HiBiT tag to structural proteins required for VLP assembly (49–51).

Although the present study focused primarily on applications for NMAb development, we anticipate that HiBiT-PsVLP bioassays could be implemented for other antiviral modalities. NAb titers are considered an important correlate of protection against many viruses, and a similar HiBiT-tagged VLP approach was recently described for screening post-vaccination sera against SARS-CoV-2 (52, 53). In contrast, our HiBiT-PsVLP system omits the HIV-1 *pol* gene for improved biosafety and employs a streamlined, homogenous workflow well suited to high-throughput screening. The proven flexibility of our platform and its ability to assess ADE provide additional advantages for vaccine research. HiBiT-PsVLP bioassays could also aid in developing small-molecule entry inhibitors targeting viral glycoproteins or essential host factors.

Beyond antiviral drug development, we envision key applications for HiBiT-PsVLP technology in basic virology research. In our assays, luminescence kinetics generally varied in ways consistent with known entry pathways—for example, signals appeared more rapidly for viruses that fuse at the plasma membrane compared to those requiring endocytosis and intracellular trafficking. Such pathway-dependent differences suggest that HiBiT-PsVLPs could provide a biologically relevant system for probing entry mechanisms (e.g., receptor usage, endocytic dependence, pH sensitivity) in a manner similar to traditional pseudoviruses. Given their non-replicative nature, HiBiT-PsVLPs could also serve as a safe platform for screening glycoprotein mutations that alter receptor usage and host range, without many of the gain-of-function concerns associated with replication-competent systems.

## MATERIALS AND METHODS

### Cell lines and cell culture

293T (CRL-3216) and THP-1 (TIB-202) cell lines were purchased from the American Type Culture Collection (ATCC). 293T cells were cultured in Dulbecco’s Modified Eagle Medium (DMEM; Gibco, 11995) supplemented with 10% fetal bovine serum (FBS; Avantor Seradigm, 89510-194), 1 mM sodium pyruvate (Gibco, 11360070), and 1× MEM non-essential amino acids (Gibco, 11140050). THP-1 cells were cultured in RPMI 1640 medium (Gibco, 22400) supplemented with 10% FBS.

### Plasmids

Sequence encoding the HiBiT peptide (VSGWRLFKKIS) was appended to the 3′ end of an HIV-1 Gag ORF codon-optimized for Rev-independent expression (54). This Gag^HiBiT^ ORF was inserted into a mammalian expression vector under control of the Cytomegalovirus (CMV) immediate enhancer/chicken β-actin (CAG) promoter. LgBiT and HaloTag-LgBiT were encoded by LgBiT Expression Vector (Promega, N268A) and CMV HaloTag-LgBiT Vector (Addgene, 236917), respectively. PCSK9^HiBiT^, human ACE2, and human CD4 ORFs were inserted into CAG or CMV promoter-driven vectors. A single ORF encoding human CCR5 and human CXCR4 separated by a P2A self-cleaving peptide linker was cloned into a CMV promoter-driven vector. Human TMPRSS2^FLAG^ ORF was inserted into an EF1α promoter-driven vector. Human codon-optimized ORFs encoding viral entry proteins were cloned into CAG or CMV promoter-driven vectors. The endodomains of several viral glycoproteins were truncated to improve pseudotyping based on previous reports (Table 2). Antibody expression constructs were generated via insertion of the heavy and light chain variable sequences of imdevimab or mAb114 into vectors encoding the human IgG1 heavy and lambda (imdevimab) or kappa (mAb114) light chain constant regions.

**TABLE 2:** Viral entry proteins used in this study^*a*^

Source virus	Strain, variant, or isolate	Entry protein	NCBI protein sequence ID	Length of endodomain truncation (amino acids)^*b*^
EBOV	Mayinga, 1976	GP	AHL68679.1	–
HIV	BaL	Env	ABC55874.1	146
	JRFL	Env	AAB05604.1	146
	NL4-3	Env	AAA44992.2	146
LASV	Josiah	GPC	NP_694870.1	–
MARV	Angola, 2005	GP	Q1PD50.1	–
NiV	Malaysia	F	NP_112026.1	22
		G	NP_112027.1	–
SARS-CoV-2	B.1	S	CAD0240757.1	18
SEBOV	Gulu, 2000	GP	YP_138523.1	–
VSV	Indiana	G	ABD73123.1	–

^*a*^ Where indicated, the specified number of amino acids was removed from the C-terminal endodomain to enhance pseudotyping.

^*b*^ – indicates no truncation.

### Antibodies and reagents

Antibodies used for immunoblotting were as follows: anti-HiBiT monoclonal antibody (Promega, N7200), anti-SARS Spike protein antibody (Novus Biologicals, NB100-56578), anti-GAPDH antibody (Santa Cruz, SC-47724), anti-Mouse IgG (H+L) HRP conjugate antibody (Promega, W4021), and anti-Rabbit IgG (H+L) HRP conjugate antibody (Promega, W4011).

The following antibodies were purchased from Biolegend and used for flow cytometric and fluorescence-activated cell sorting (FACS) analyses: PE anti-DYKDDDDK (FLAG) (637309), PE anti-human CD4 (317410), Alexa Fluor 488 anti-human CD195 (CCR5) (359103), APC anti-human CD184 (CXCR4) (306509), Brilliant Violet 421 anti-human CD64 (305020), and FITC anti-human CD32 (303204). For ACE2 detection, Human ACE2 antibody (R&D Systems, AF933) was used in combination with Alexa Fluor 647 AffiniPure Donkey Anti-Goat IgG secondary (Jackson ImmunoResearch, 705-605-147).

Antibodies used in neutralization assays are listed in Table 3. F(ab′)_2_ fragment of anti-Ebola clone KZ52 was generated using the Pierce (Fab′)_2_ Micro Preparation Kit (Thermo Scientific, 44688). Camostat mesylate was purchased from Sigma (SML0057).

**TABLE 3:** Antibodies used for neutralization assays

Target	NMAb name	Clone	Vendor	Catalog no.
SARS-CoV-2 Spike	Bamlanivimab biosimilar	LY-CoV555	Invivogen	srbdc5-mab1
	Casirivimab biosimilar	REGN10933	Invivogen	10497-44-01
	Cilgavimab biosimilar	AZD1061/COV2-2130	Invitrogen	MA5-42313
	Etesevimab biosimilar	LY-CoV016	Invivogen	srbdc6-mab1
	Imdevimab biosimilar	REGN10987	Invivogen	10498-44-01
	Sotrovimab biosimilar	S309	Invitrogen	MA5-42316
	Tixagevimab biosimilar	AZD8895/COV2-2196	Invitrogen	MA5-42312
	Anti-SARS-CoV-2 S protein S1	414-1	Biolegend	938702
	Anti-SARS-CoV-2 S protein S1	415-6	Biolegend	938601
	Anti-Spike protein	4A8	Absolute Antibody	Ab02743-10.0
	Spike neutralizing antibody	G10xA5	BPS Bioscience	101327
	Spike neutralizing antibody, rabbit Mab	R001	SinoBiological	40592-R001
HIV gp120	Anti-HIV-1 gp120 protein	VRC01	BEI Resources	ARP-12033
EBOV GP	Anti-Ebola surface glycoprotein	KZ52	Absolute Antibody	Ab00690-10.0
	Cosfroviximab	c13C6-FR1	MedChemExpress	HY-P99832
SEBOV GP	Mouse anti-SUDV GP	16F6	IBT Bioservices	0280-001
MARV GP	Anti-Marburg GP macaque-derived chimeric mAb	N-125	IBT Bioservices	0203-028
LASV GPC	Anti-glycoprotein complex	37.7H	Absolute Antibody	Ab01102-10.0
NiV F	Anti-F glycoprotein	12B2	Absolute Antibody	Ab02792-10.0

### Transfection and stable cell line generation

293T-derived target cell lines were generated via plasmid transfections using ViaFect Reagent (Promega, E4981), followed by antibiotic selections and clonal isolation via FACS. LgBiT expression was confirmed by flow cytometry and/or by detecting LgBiT activity using the Nano-Glo HiBiT Lytic Detection System (Promega, N3040) with recombinant HaloTag-HiBiT protein (Aldevron). Expression of all other transgenes was confirmed via flow cytometry.

THP-1 cells (ATCC TIB-202) were electroporated with HaloTag-LgBiT expression vector using Ingenio Electroporation Reagent (Mirus, MIR50114) with a GenePulser Xcell (Bio-Rad). Following antibiotic selection, cells were sorted for LgBiT expression via HaloTag labeling and FACS. CRISPR RNAs (crRNAs) targeting CD64 and CD32 (Integrated DNA Technologies; IDT) were duplexed with tracrRNA (IDT, 1072533) and incubated with Cas9 nuclease (IDT, 1081059) to yield ribonucleoprotein (RNP) complexes. RNPs were electroporated into the THP-1/HaloTag-LgBiT pool using Ingenio Reagent with a Nucleofector system (Lonza). Cell pools with complete knockout of FcγRI, FcγRII, or both were obtained via FACS. Final knockout phenotypes were confirmed by flow cytometry.

### Flow cytometry

Cells were washed with phosphate-buffered saline supplemented with 2% FBS, labeled with primary antibodies for 30 min on ice, and washed twice with PBS + 2% FBS. Where necessary, cells were labeled with secondary antibody for 30 min on ice, followed by two additional rounds of washing. For detection of HaloTag-LgBiT, cells were labeled with Janelia Fluor 646 HaloTag Ligand (Promega GA1121) for 30 min at 37°C and washed once with PBS + 2% FBS. Samples were analyzed on a Fortessa X-20 flow cytometer (BD), and data were analyzed using FlowJo Software (BD).

### Production and characterization of HiBiT-PsVLPs

HiBiT-PsVLPs were generated via transient transfection of 293T producer cells using ViaFect Reagent with Gag^HiBiT^ expression vector and plasmid(s) encoding the specified viral glycoprotein(s). Transfection Carrier DNA (Promega, E4881) was substituted for plasmids encoding viral glycoproteins to generate Bald HiBiT-VLP controls. Culture supernatants were collected 48 to 72 h post-transfection, centrifuged at 1,000 × *g* for 10 min at 4°C, and passaged through 0.45 µm PVDF filters (Merck Millipore, SLHVR33RS). HiBiT-PsVLP preps were used immediately or stored at −80°C. HiBiT packaging was assessed using the Nano-Glo HiBiT Lytic Detection System and the Nano-Glo HiBiT Extracellular (i.e., non-lytic) Detection System (Promega, N2421). HiBiT content per particle, expressed as the ratio of HiBiT to HIV-1 p24 protein, was determined using the Nano-Glo HiBiT Lytic Detection System in parallel with the Lumit p24 Immunoassay (Promega, CS2039B25). Ratios were normalized to a reference batch of Bald HiBiT-VLPs.

For immunoblotting, culture supernatants and 293T producer cells were harvested following HiBiT-PsVLP production and treated with mammalian lysis buffer (Promega, G9381) containing protease inhibitors (Promega, G6521). Protein concentrations in cell lysates were quantified using a BCA assay (ThermoFisher, 23227). Samples were separated via SDS-PAGE on a precast 4%–20% Criterion TGX protein gel (Bio-Rad, 5671094) and transferred onto nitrocellulose membranes using the iBlot 2 Dry Blotting System (Invitrogen). Membranes were blocked with tris-buffered saline containing 0.1% Tween-20 (TBST) and 5% non-fat dry milk for 1 h at room temperature, washed with TBST, and incubated overnight with primary antibodies at 4°C. After washing with TBST, membranes were incubated with secondary antibodies for 1 h at room temperature, washed again with TBST, and incubated for 1 min with ECL Western Blotting Substrate (Promega, W1001). Blots were imaged on a ChemiDoc MP Imaging System with Image Lab software (Bio-Rad).

For electron microscopy, HiBiT-PsVLPs were produced as described above and concentrated using Amicon Ultra-15 Centrifugal Filters with a 100 kDa molecular weight cut-off (Merck Millipore, UFC9100008). Concentrates were loaded onto a 20% sucrose cushion, centrifuged at 112,000 × *g* for 70 min at 10°C using an Optima TLX Ultracentrifuge (Beckman Coulter), and pellets were resuspended in PBS. Samples were negatively stained with Nano-W (Nanoprobes) on a formvar-coated 300 mesh Cu Thin-Bar grid (EMS), coating side down using a two-step method. Samples were viewed on a Philips CM120 transmission electron microscope at 80 kV and documented with an AMT BioSprint12 digital camera at the Electron Microscope Core of the University of Wisconsin.

### HiBiT-PsVLP entry assays

For time-course analyses, the indicated target cells were incubated for 1 h at 37°C, 5% CO_2_ in DMEM + 0.5% FBS containing Nano-Glo Vivazine Substrate (Promega, N2581) in 96-well white flat-bottom assay plates (Corning). Bio-Glo-NB DrkBiT Peptide (Promega), a membrane-impermeable peptide that binds free LgBiT to prevent extracellular NanoBiT complementation, was added during this pre-incubation step to eliminate background luminescence unrelated to HiBiT-PsVLP entry. HiBiT-PsVLPs were overlaid onto target cells, and luminescence was measured at the indicated time points using a GloMax Discover Microplate Reader (Promega). Specificity of SARS-CoV-2 S HiBiT-PsVLP entry was assayed by overlaying HiBiT-PsVLPs onto 293T cells expressing LgBiT with or without ACE2 and TMPRSS2. Assay plates were incubated at 37°C, 5% CO_2_ for 4 h, followed by the addition of Bio-Glo-NB Live Cell Reagent (Promega), further incubation at 37°C for 15 min, and luminescence measurement. Data were analyzed using GraphPad Prism Software.

### Neutralization assays

Frozen stocks of HiBiT-PsVLPs were thawed at 37°C and incubated with serial mAb titrations in DMEM + 0.5% FBS for 30 min at 37°C, 5% CO_2_ in 96-well white flat-bottom assay plates. Target cells were incubated with Bio-Glo-NB DrkBiT Peptide in DMEM + 0.5% FBS for 15 min at 37°C, 5% CO_2_, and added to assay wells containing the HiBiT-PsVLPs and mAbs. Mock control wells containing target cells alone were included on each plate. Assay plates were incubated at 37°C, 5% CO_2_. Incubation times were selected based on glycoprotein-specific entry kinetics of HiBiT-PsVLPs: 3 h for SARS-CoV-2 S, 4 h for HIV Env, 5 h for LASV GPC and NiV-F/G, 22 h for MARV GP and SEBOV GP, and 24 h for EBOV GP. Luminescence was determined by adding the Bio-Glo-NB Live Cell Reagent at assay endpoints, as described above. Baseline-corrected luminescence was determined by subtracting the average RLU value measured in Mock control wells. Percent neutralization was calculated as 100 × (1 − [RLU_+inhibitor_/RLU_no inhibitor_]). 4-PL curves were plotted using GraphPad Prism Software. Neutralization assays in 384-well white flat-bottom assay plates (Corning) were performed as above, albeit with reduced volumes of each assay component.

To assess neutralization by transiently expressed mAbs, 293T cells were transfected with IMD- or mAb114-huIgG1 expression plasmids. Supernatants were collected 72 h post-transfection, and neutralizing activity was assayed as above. For TMPRSS2 inhibition, a serial titration of camostat mesylate was prepared in DMEM + 0.5% FBS and incubated with target cells and Bio-Glo-NB DrkBiT Peptide for 25 min at 37°C, 5% CO_2_. HiBiT-PsVLPs were then added, and the remainder of the assay was performed as above.

The SARS-CoV-2 Surrogate Virus Neutralization Test (Genscript, L00847-A) was performed according to the manufacturer’s protocol. NMAb IC_50_ values derived from the sVNT were plotted against those from HiBiT-PsVLP bioassays, and a linear correlation analysis was performed using GraphPad Prism Software.

### SARS-CoV-2 HiBiT-PsVLP bioassay qualification

Assay linearity was determined by two separate analysts across three independent assays each. For each assay, titrations of cilgavimab biosimilar were prepared at 50%, 70%, 100%, 150%, or 200% potency relative to a reference titration. JMP software (JMP) was used to plot 4-PL curves, test parallelism (F test), and determine relative potency values. Measured relative potency values were plotted against expected values using GraphPad Prism Software, and data were fitted with a linear regression. The mean and standard deviation of measured potency values were used to calculate accuracy (i.e., %recovery) and intermediate precision. Repeatability was determined from six independent titrations of cilgavimab biosimilar analyzed in a single experiment. For stability-indicating studies, cilgavimab biosimilar was incubated at 4°C or at 65°C for the indicated lengths of time before performing the HiBiT-PsVLP neutralization bioassay.

### Antibody-dependent enhancement assays

MAbs or F(ab′)_2_ fragments were prepared in RPMI + 0.5% FBS and added to EBOV GP HiBiT-PsVLPs in 96-well white flat-bottom assay plates. THP-1/HaloTag-LgBiT target cell lines were incubated with Bio-Glo-NB DrkBiT Peptide in RPMI + 0.5% FBS for 15 min at 37°C, 5% CO_2_, then added to assay wells. Assay plates were incubated for 24 h at 37°C, 5% CO_2_, and luminescence was determined by adding the Bio-Glo-NB Live Cell Reagent at the assay endpoint, as described above. Fold changes in luminescence were calculated as RLU_+antibody_/RLU_no antibody_. Data were analyzed using GraphPad Prism Software.

## Data Availability

The authors declare that the data supporting the findings of this study are available within the article and supplemental material.

## SUPPLEMENTAL MATERIAL

Supplemental figures jvi.00991-25-s0001.docx Figures S1 to S5.

ASM does not own the copyrights to Supplemental Material that may be linked to, or accessed through, an article. The authors have granted ASM a non-exclusive, world-wide license to publish the Supplemental Material files. Please contact the corresponding author directly for reuse.
