# Supplementary material for: A HiBiT-tagged pseudovirus-like particle platform for safe, rapid quantification of virus neutralization and antibody-dependent enhancement
Source: J Virol. 2025 Oct 8;99(11):e00991-25. doi: 10.1128/jvi.00991-25 (PMC12645950; doi:10.1128/jvi.00991-25)
Supplement: Supplemental figures — Figures S1 to S5. [file jvi.00991-25-s0001.docx]

**Supplemental Material**


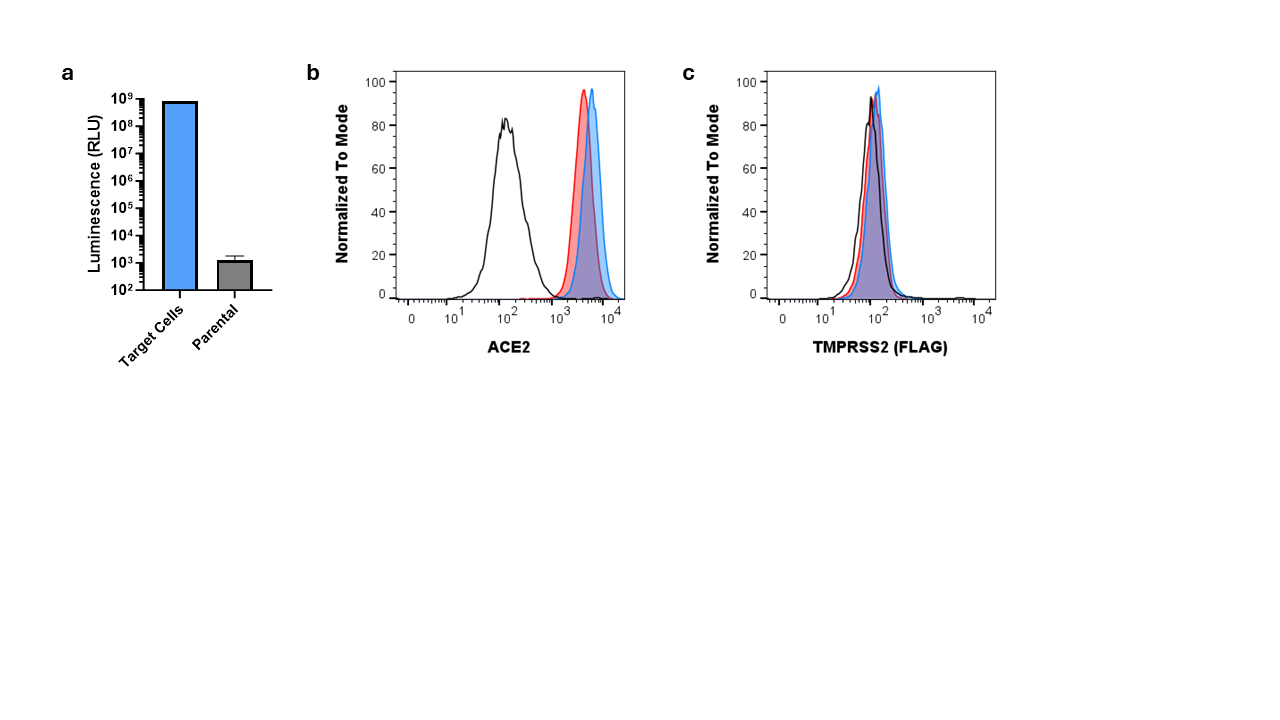


**Figure S1. SARS-CoV-2 HEK293T(LgBiT) Target Cells.** (a) LgBiT activity in freshly cultured SARS-CoV-2 HEK293T(LgBiT) Target Cells vs. parental 293T control cells. (b) ACE2 expression and (c) TMPRSS2^FLAG^ expression in parental 293T cells (black), freshly cultured SARS-CoV-2 HEK293T(LgBiT) Target Cells (blue), and Thaw-and-Use SARS-CoV-2 HEK293T(LgBiT) Target Cells (red).


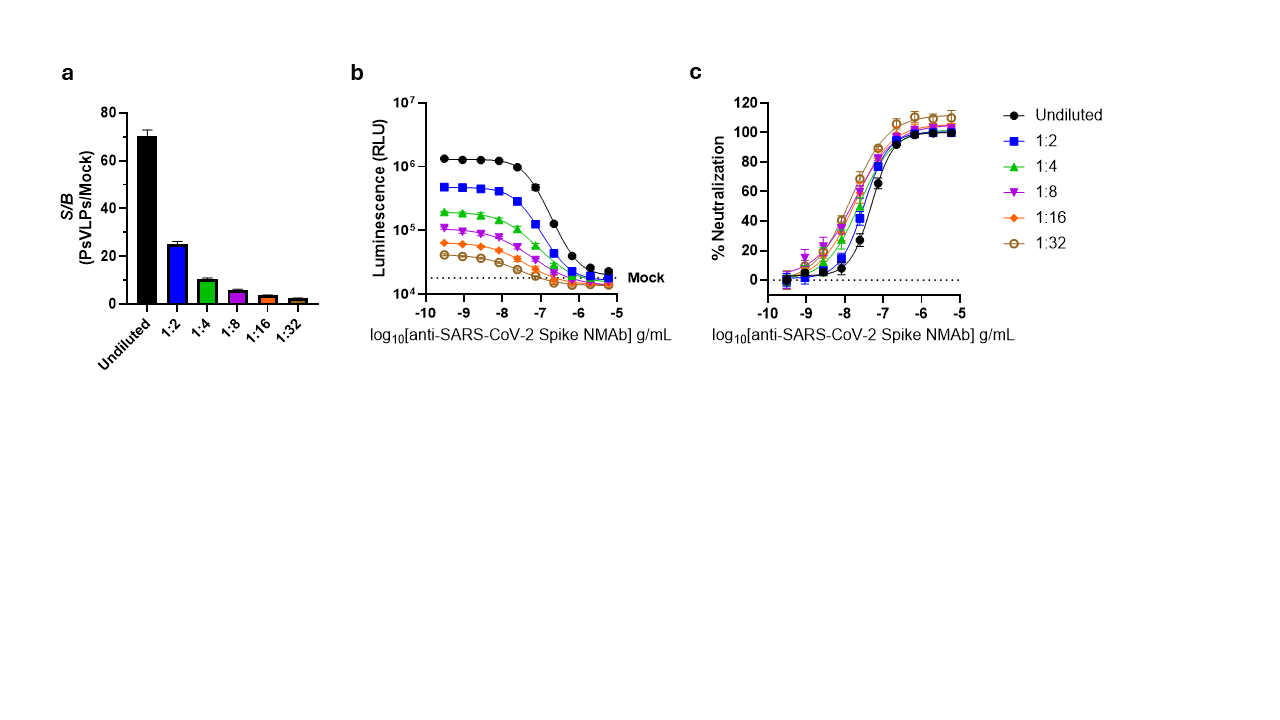


**Figure S2. Titrating SARS-CoV-2 S HiBiT-PsVLPs for neutralization assay performance.** (a) Signal-to-background (*S/B*) ratios for SARS-CoV-2 S HiBiT-PsVLPs added to SARS-CoV-2 HEK293T(LgBiT) Target Cells at increasing dilutions. *S/B* ratios were calculated as the RLUs from wells containing HiBiT-PsVLPs and Target Cells divided by RLUs from wells containing Target Cells alone (Mock). (b-c) Neutralization of SARS-CoV-2 S HiBiT-PsVLPs at different dilution factors. (b) Raw luminescence values. Dashed line represents luminescence in Mock control wells. (c) Percent neutralization values.


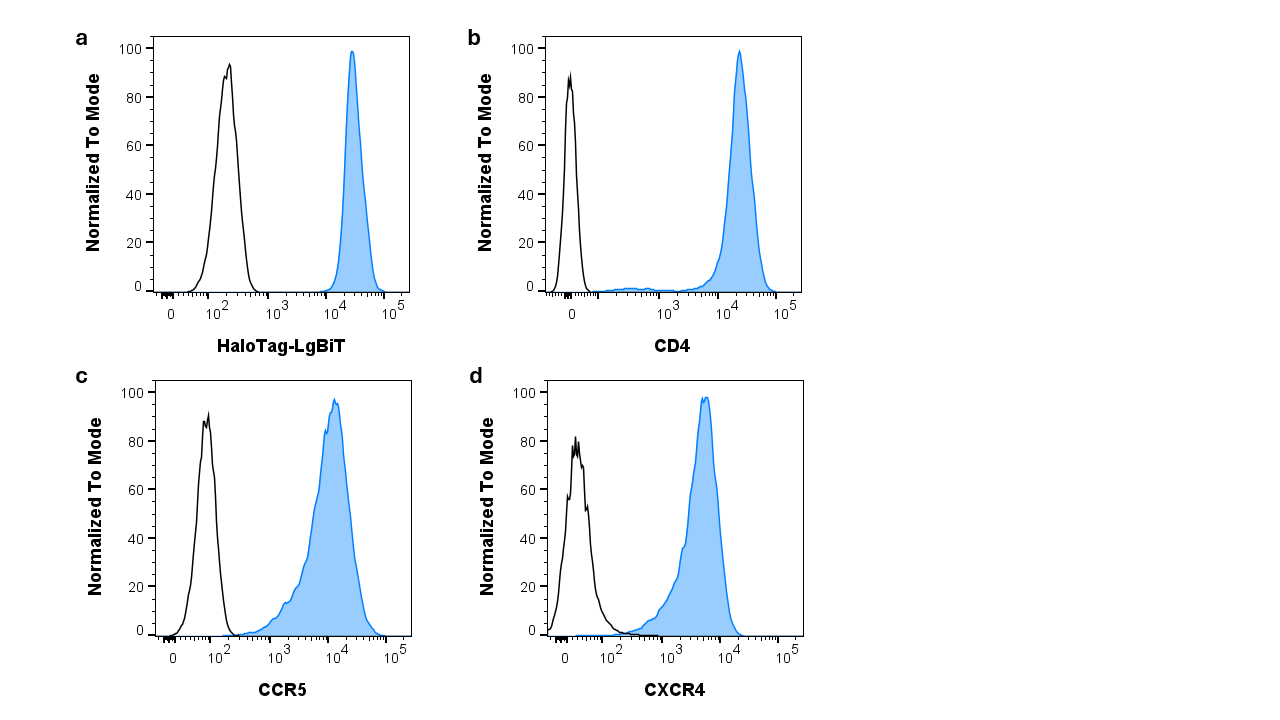


**Figure S3. HIV HEK293T(LgBiT) Target Cells.** Expression of (a) HaloTag-LgBiT, (b) CD4, (c) CCR5, and (d) CXCR4 in parental 293T cells (black) vs. HIV HEK293T(LgBiT) Target Cells (blue) as detected by flow cytometry.


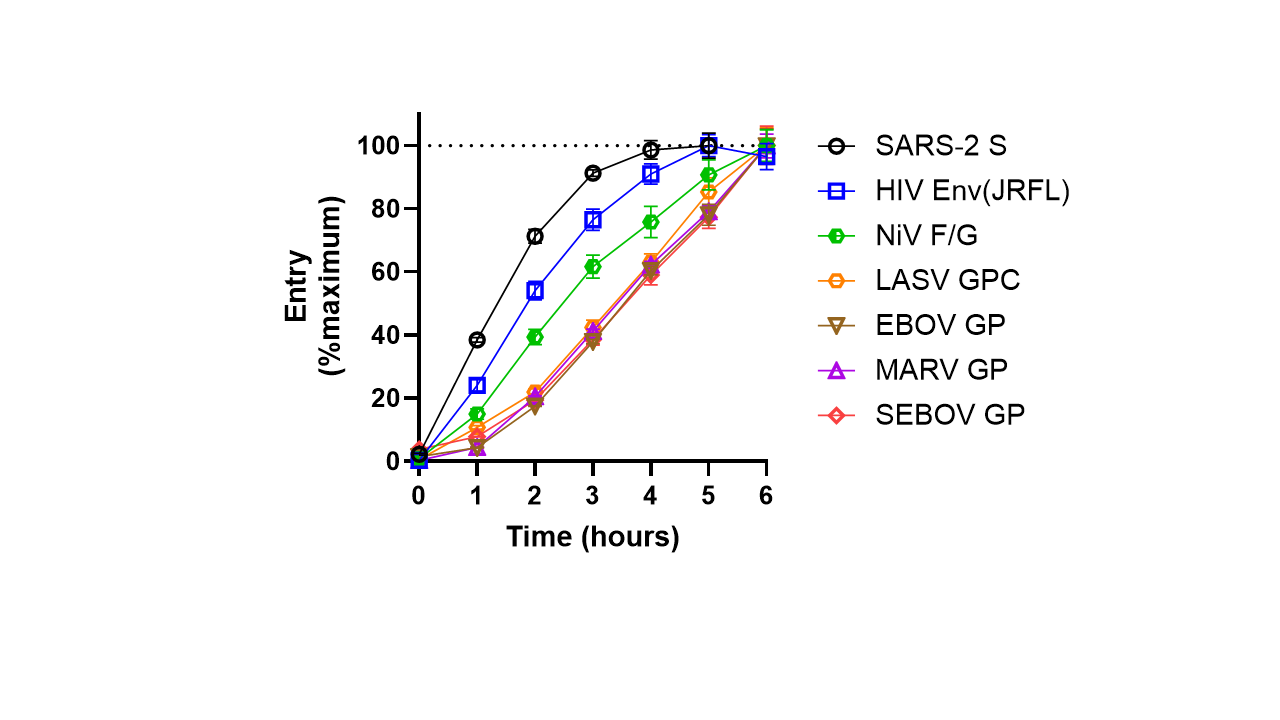


**Figure S4. Entry kinetics for the indicated HiBiT-PsVLPs.** Data are from the same experiments shown in Figures 2b, 6a, 7a, 7e, and 7g. Values represent the percentage of the maximum observed entry signal for each HiBiT-PsVLP across timepoints.


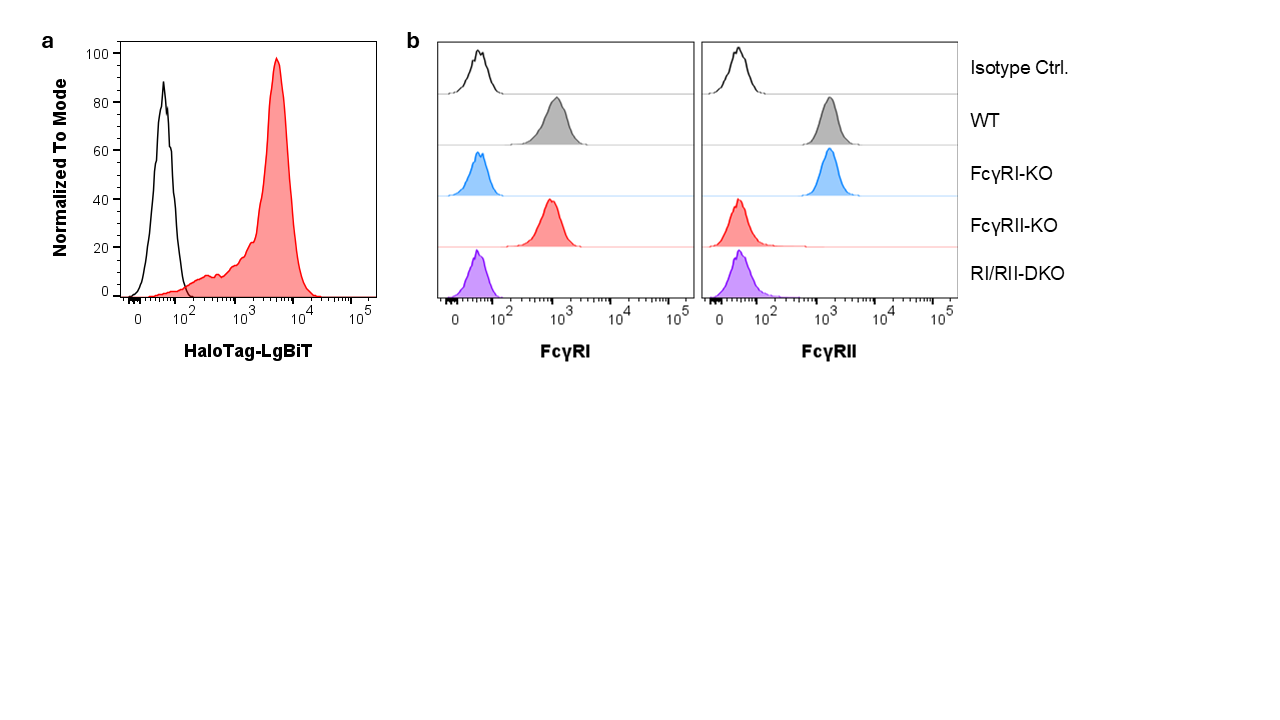


**Figure S5. THP-1 cell-lines for assessment of ADE.** (a) Flow cytometry for HaloTag-LgBiT expression in parental THP-1 cells (black) vs. THP-1/HaloTag-LgBiT cells (red). (b) Flow cytometry for FcγRI and FcγRII expression in wild-type (WT) vs. CRISPR-KO THP-1/HaloTag-LgBiT cell-lines. DKO, double-knockout.
